# A Comprehensive Analysis of the Nutritional Composition of Plant-Based Drinks and Yogurt Alternatives in Europe

**DOI:** 10.3390/nu15153415

**Published:** 2023-07-31

**Authors:** Elphee Medici, Winston J. Craig, Ian Rowland

**Affiliations:** 1 Nutrilicious Ltd., London N2 0EF, UK; 2 Center for Nutrition, Healthy Lifestyles and Disease Prevention, School of Public Health, Loma Linda University, Loma Linda, CA 92354, USA; 3 Hugh Sinclair Human Nutrition Unit, Department of Food and Nutritional Sciences, University of Reading, Reading RG6 6DH, UK; i.rowland@reading.ac.uk

**Keywords:** plant-based alternatives, calcium, iodine, vitamin D, vitamin B12, sugars, protein, Europe, food-based dietary guidelines, healthy population

## Abstract

Concerns for human and planetary health have led to a shift towards healthier plant-based diets. Plant-based dairy alternatives (PBDA) have experienced exponential market growth due to their lower environmental impact compared to dairy products. However, questions have arisen regarding their suitability as dairy substitutes and their role in food-based dietary guidelines (FBDG). Our study aimed to analyse the nutritional profiles of leading PBDA across Europe and compare them with their dairy counterparts. We examined the nutritional profiles of 309 unflavoured PBDA representing the European market leaders, including 249 plant-based drinks (PBD) and 52 plant-based alternatives to yogurt (PBAY). PBD and PBAY, excluding coconut varieties, were low in saturated fat (<1 g per serving). Seventy percent of PBDA were unsweetened, and most had sugar levels comparable to dairy. Except for soya varieties, PBDA protein levels were lower than dairy. Organic PBDA lacked micronutrients due to legal restrictions on fortification. Among non-organic PBDA, 76% were fortified with calcium, 66% with vitamin D, and 60% with vitamin B12. Less than half were fortified with vitamin B2, and a few with iodine (11%) and vitamin A (6%). PBAY were less frequently fortified compared to PBD. PBDA displayed a favourable macronutrient profile despite lower protein levels, which would be compensated for by other protein-dense foods in a usual mixed diet. Enhancing fortification consistency with dairy-associated micronutrients would address concerns regarding PBDA’s integration into FBDG. Our analysis supports the inclusion of fortified PBDA in environmentally sustainable FBDG for healthy populations.

## 1. Introduction

The impact of our current food system on human health, the environment, and animal welfare is a significant concern globally [1,2,3,4,5]. The negative consequences of the current food system include the continued growth of non-communicable and zoonotic diseases, global warming, land use change, biodiversity loss, eutrophication, and excessive withdrawals of freshwater resources for agriculture [2,4,6]. To address these issues, the international community is actively working towards creating a more sustainable food system. A key aspect of this effort involves shifting away from the current reliance on beef and dairy cattle agriculture, which is the largest contributor to our diet-related environmental burden [1,2,4,7,8]. The consensus is to promote diets that include more plant-based foods and less animal-based foods, especially meat and dairy [1,2,4,6,9].

Plant-based dairy alternatives (PBDA), such as plant-based drinks (PBD) and plant-based alternatives to yogurt (PBAY), have emerged as a promising solution to promote more sustainable dietary patterns. Compared with their dairy counterparts, PBDA and their base ingredients have a significantly lower environmental burden, including greenhouse gas emissions, eutrophication, land use, and water acidification [10,11].

Products can be classified as PBDA based on either one of two criteria: Firstly, if they provide nutrients such as protein, calcium, and other vitamins and minerals closely resembling their dairy counterparts. Alternatively, PBDA products can be considered if they can be used and consumed in a similar manner to dairy, even if their nutritional profile differs. PBDA are based on a variety of plant ingredients, including legumes, grains, nuts, and seeds.

National food-based dietary guidelines (FBDG) are starting to include unflavoured, fortified soya-based PBD and PBAY alongside dairy [12,13,14,15,16,17,18,19]. In the UK, any unsweetened calcium-fortified PBD can be used as a main drink for children older than 12 months [20].

Integrating PBDA into everyday eating patterns is becoming easier, as they help to leverage factors that encourage changes in dietary behaviour [21,22,23]. These factors include improved accessibility, usage familiarity, and social normalisation: PBDA products are readily available in most supermarkets [24], making them easily accessible to consumers; they can be used in the same way as dairy products, eliminating the need for consumers to learn new skills or disrupt their usual routines; and the placement of PBDA alongside their dairy counterparts in supermarket aisles and their increased presence in the food environment has helped make them socially acceptable and normalised. As a result, consumers find it easier to embrace PBDA without feeling they are making a drastic or unconventional change.

In recent years, the market for PBDA has surged globally [25] and across Europe [24,26]. The global PBD market is estimated to be worth USD 12.1 to USD 18.5 billion and projected to reach in excess of USD 24 billion by 2025 [27,28,29]. In Europe, the overall PBDA market is estimated to be worth EUR 3 billion, rising to EUR 5 billion by 2025, [24] with unflavoured PBD accounting for the majority of the market at EUR 2.2 billion in 2019/20 [30,31,32]. PBAY make up a smaller percentage of the market, but they are also fast-growing, with a value of EUR 627 million in 2019/2020 [31,32]. Oat, soya, and almond make up the majority of the PBD market, with oat showing the biggest growth. In contrast, soya and coconut dominate the PBAY market [32].

The growing popularity of PBDA can be attributed to a variety of factors, including the rise of the “flexitarian” diet, increasing concerns for animal welfare, and a growing interest in sustainable eating, particularly among younger generations and millennials [33,34]. A recent survey conducted in Europe found that 17% of individuals had reduced their dairy intake, while 24% had added PBDA to their shopping baskets in an effort to follow a more sustainable diet [33]. A significant driver is consumers’ search for health solutions, including gut health and intolerances [35]. Interestingly, many consumers seem to complement their dairy intake with PBDA rather than completely displacing dairy in their diet [25,36,37]. The current decline in cow’s milk consumption can only be partially attributed to the growing popularity of PBD. A number of authors have highlighted that the decline in unflavoured cow’s milk consumption has been accompanied by a simultaneous and significant increase in the consumption of flavoured dairy drinks, yogurts, and cheese [28,38,39].

While it is widely recognised that switching to PBDA can have significant environmental benefits, there are concerns about their nutritional value, which has raised doubts about their appropriateness for inclusion within FBDG. Several studies have assessed the nutritional composition of PBDA available on the market. The majority of studies conducted so far have primarily assessed the nutritional profile of PBD [27,38,39,40,41,42,43,44,45]. Comparatively, fewer studies have investigated PBAY [46,47,48,49]. Typically, the nutritional profile of cow’s milk is used as the benchmark for comparing the nutrient profile of PBD.

The primary nutrient of focus of previous research has been the protein and amino acid profile of PBDA, along with other macronutrients; salt (sodium chloride); and micronutrients typically associated with dairy products, such as calcium, vitamin B12, and vitamin D for countries that fortify. The majority of these studies indicate that, with the exception of certain soya and pea protein-based products, the protein content and quality of PBDA are significantly lower compared to dairy milk, in many cases providing no more than 1 g of protein per 100 mL [38,39,40,41,42,43,44,45]. On the other hand, PBD and PBAY have been found to have lower energy values and saturated fat levels (with the exception of coconut-based products) compared with their dairy counterparts [27,38,41,42,43,46,49,50]. Inconsistency in the macronutrient profiles of PBD across different ingredient bases and brands is commonly observed.

Fortification is often practised to increase the micronutrient content of PBDA; however, there is a lack of consistency among PBD and PBAY concerning micronutrient fortification. While PBD and PBAY are often fortified with calcium, fortification with other crucial micronutrients present in dairy, such as iodine (important for some European countries) and vitamins B2, B12, and D, is infrequent and inconsistent [38,39,41,45,46,51]. Fortification has been highlighted as important in addressing the micronutrient disparities between PBDA and their dairy counterparts, with the aim of establishing their adequacy as viable substitutes for dairy products [38,41].

The aim of our research was to investigate the nutritional composition of currently available PBD and PBAY in comparison to their dairy counterparts across Europe, and in so doing, assist decision-making when considering the role of PBDA within sustainable FBDG. As FBDG exclude both dairy and PBDA that are flavoured, our investigation solely focused on plain unflavoured PBD, PBAY, and dairy.

## 2. Materials and Methods

### 2.1. PBDA, PBD, and PBAY Definitions

For the purposes of our study, we considered the current positioning of PBDA within the dairy section of stores and the fact that citizens use PBDA in place of dairy irrespective of nutrition profile.

### 2.2. Sample Selection

Three types of PBDA currently available in the European market were included:

1. PBD. Plant-based drinks are manufactured by extracting a base plant ingredient in water and homogenising the fluid to mimic the appearance and usage of dairy milk.
2. PBAY. PBD which have been fermented using live-active cultures with or without the addition of thickeners and emulsifiers to mimic the texture and sensory properties of dairy yogurt.
3. PBAY Greek-style (PBAY GS). In addition to PBAY, which mimic standard dairy yogurt, new PBAY varieties with a higher protein content and mimicking the thicker consistencies of Greek-style dairy yogurts were also included.

To ensure that our sample of PBDA accurately represented the European market, we utilised 2022 Mintel datasets to determine the sales market share value (as a moving annual total) by brand for PBD, PBAY, and PBAY GS in Europe [52]. From these datasets, we selected the top-selling brands, which accounted for around 59% of the PBD and 73% of the PBAY/GS market. Additionally, we discovered that private labels (retailer brands) made up 30% of the remaining PBD and 17% of the PBAY market share. To account for this, we identified private labels from the largest food retailers in seven European countries (Belgium, France, Germany, Spain, Sweden, The Netherlands, and the UK).

Dairy comparisons: as PBD, in general, are low in fat, and FBDG recommend lower fat dairy milk in place of full-fat, we selected dairy milk with a low-fat profile and as close to the ‘semi-skimmed’ classification of 1.8% fat as the comparison.

Similarly, for yogurts, we included very low-fat (<0.1% fat) and low-fat (≤1.5%) varieties. For Greek-style yogurt comparisons, there are significant differences in fat content across Europe. We, therefore, included all types where nutritional information was made available.

### 2.3. Nutritional Relevance of PBDA for the General Population

With regard to interpreting the nutritional adequacy of PBDA, we have prioritised public health in our review, with the general population as the target audience, i.e., children over 2 years and adults in general good health consuming a mixed diet and meeting their protein and energy needs.

### 2.4. Study Eligibility Criteria

Included in our study: Plain (unflavoured) varieties of PBDA and plain (unflavoured) dairy comparisons were included, where all the required information was available online. We defined plain unflavoured products as those that did not contain fruit or other added flavours, such as chocolate and vanilla. Plain unflavoured products include both unsweetened and sweetened (i.e., with added sugars). Unique brand recipes were included only once in the analysis, irrespective of how many countries they appeared in.

Excluded from our study: Full-fat dairy milk and yogurts (excluding Greek-style); flavoured PBDA and flavoured dairy (e.g., fruited, chocolate, or vanilla); products where full nutritional information could not be retrieved; added value recipes that were significantly skewed for specific nutrients (e.g., high-protein drinks; drinks specifically targeted for children under 3 years of age with added nutrients not normally associated with dairy milk, e.g., iron and vitamin C; pro- and prebiotic varieties). With regard to interpreting the nutritional adequacy of PBDA, we excluded population groups with specific and heightened nutritional needs and/or compromised dietary intakes who are likely to require specialised dietetic support and disproportionately rely on milk and dairy products, e.g., infants, elite athletes, elderly with compromised health, and those requiring clinical support.

### 2.5. Data Collection

For the market-leading brands identified, we accessed the nutritional information from the manufacturers’ websites, and for the private labels, from the supermarket websites. If the primary website search did not provide adequate nutritional information, the nutritional database Open Food Facts was used [53].

#### 2.5.1. General Characteristics

General characteristics included the brand, product name, the country or countries they were available, the base plant ingredient/s used, the presence or absence of free and added sugars (as categorised by the European Food Safety Authority (EFSA) [54]), and whether they were labelled as organic (or ‘bio’).

The ingredient base of the products was categorised as “single ingredient base” or “mixed ingredient base” (if based on more than one plant ingredient).

#### 2.5.2. Nutrition Data

Nutrition information (including fortification) for dairy milk and yogurts (standard and Greek-style) was accessed from the official national food databases of 11 European countries (Belgium [55], Denmark [56], Finland [57], France [58], Germany [59], Norway [60], Spain [61], Sweden [62], Switzerland [63], The Netherlands [64], and the UK [65]).

The following macronutrient data were collected: kilojoules (kJ), kilocalories (kcal), fat in grams (g), saturated fat (g), carbohydrates (g), total sugars (g), fibres (g), and protein (g). Additionally, salt (g) was also noted, and in a few cases where sodium values in milligrams (mg) were provided, we converted to g of salt.

Micronutrient information most commonly associated with dairy milk and yogurts were collected: calcium (mg), iodine (mcg), vitamins D (mcg), B2 (mg), B12 (mcg), and A (mcg).

Extracted data were recorded in an Excel spreadsheet. Duplicate recipes appearing in multiple countries were removed.

### 2.6. Statistical Analysis

All original nutritional values per 100 mL or 100 g were converted to standard serving sizes for statistical analysis. The standard serving sizes used were 250 mL for milk and PBD (representative of the recommended daily intake by EAT Planetary Health Diet [66]) and 150 g for PBAY, PBAY GS, and dairy yogurts.

The normality of data distribution was first tested through the Shapiro–Wilk test and rejected. Descriptive statistics (median and interquartile range) of energy (kJ and kcal), macronutrients (total fats, saturated fats, carbohydrates, sugars, fibre, protein, and salt), and selected micronutrients (i.e., calcium, iodine, vitamin D, vitamin B2, vitamin B12, and vitamin A) per serving were calculated.

The characterisation was performed for all assessed food groups (i.e., PBD, PBAY, PBAY GS, milk, yogurt, and Greek-style yogurts) as a whole and also according to their main base ingredient/s and type of dairy yogurt. Energy and macronutrient content were assessed for all products. For the micronutrient profile analysis only, products certified as organic were excluded, as these are not permitted to be fortified with micronutrients by Europe Organic food regulations [67].

Comparisons between all dairy and all PBD and all dairy and all PBAY were carried out using the Mann–Whitney U test. The Student *t*-test was used for all dairy and all PBAY GS independent samples. The Kruskal–Wallis test was followed by the Dwass–Steel–Critchlow–Fligner test for multiple comparison analysis was fit to compare food products according to their main ingredient/s and type of dairy yogurt. The statistical analysis was performed through the statistical software jamovi (version 2.3.18), with the significance level set at *p* < 0.05.

In addition to comparing the micronutrient profile of non-organic products, we investigated the percentage contribution of a single serving to micronutrient dietary reference values (DRV). We used the EFSA DRV, citing the Population Reference Index (PRI) or the Adequate Intake (AI) value when the PRI was not available [68]. Values for adults (aged ≥18 years) were used, and where there was a discrepancy between genders, we selected the highest value for the two sexes (as long as it did not reach the upper tolerable limit) (see Supplementary Table S1).

## 3. Results

### 3.1. Sample Characteristics

A total of 27 brands available across 30 European countries were included in our analysis (see Supplementary Table S2). Additionally, we included 16 private (retailer) labels from the seven European countries mentioned previously (see Supplementary Table S3).

A total of 249 PBD, 52 PBAY, and eight PBAY GS were included in our analysis.

The majority of the 249 PBD were made up of a single ingredient base of either soya (23%), oat (28%), almond (20%), coconut (9%), or rice (7%). Thirty-three (13%) PBD were classified as “other”, with the majority (25) made up of a variety of mixed ingredient bases (Table 1), and eight were of a single base ingredient (Table 1).

The majority (80%) of the 52 PBAY in our analysis were made up of a single ingredient base: soya (38%), coconut (21%), oat (13%), or almond (8%). Nineteen percent (10) of PBAY were made up of mixed ingredient bases (Table 2). PBAY GS mostly had a single ingredient base: soya (*n* = 3), oat (*n* = 2), and coconut (*n* = 2). Just one PBAY GS had a mixed base of coconut, almond, soya, and fava bean protein.

Additionally, 38% of PBD and 25% of PBAY were labelled as organic. None of the PBAY GS were organic. The majority of products within our analysis were unsweetened (without added sugars): 70% of PBD, 67% of PBAY, and 75% (6) of PBAY GS.

### 3.2. Macronutrient Profile

Table 3, Table 4 and Table 5 provide an overview of the macronutrient content per serving for PBD, PBAY, and PBAY GS, respectively, alongside their dairy counterparts. Macronutrient values for each product in our analysis can be found in Supplementary Materials Tables S4–S7.

***Energy***. Rice, oat, and ‘other single and mixed base’ PBD provided the highest median values while almond and coconut PBD were the least energy dense. PBAY varied significantly between ingredient bases, with energy values ranging from 59 to 260 kcal per 150 g serving. Coconut PBAY provided significantly higher energy values (146 kcal) per serving when compared with dairy yogurts (69 kcal) and soya PBAY (71 kcal). PBAY GS had similar energy values to Greek-style dairy.

***Total fat***. PBD ranged in fat content from 0.4 to 4.4%. Rice PBD provided the lowest fat content at 2.8 g per 250 mL serving. Soya PBD provided a median of 4.5 g of fat per serving, which was significantly higher than almond, oat, rice, and dairy. Coconut PBAY was significantly higher in total fat at 11.6 g per serving compared with dairy yogurts and soya and oat PBAY (*p* < 0.001). Total fat levels were comparable between PBAY GS ingredient bases and dairy.

***Saturated fat***. Except for products with coconut as a single or mixed ingredient base, all remaining 221 PBD were low in saturated fat (0–0.9%), providing a median of 0.5 g per 250 mL serving, which was significantly lower than low-fat dairy milk. Coconut PBD were comparable to dairy and higher than soya, rice, and other/mixed base PBD. A similar observation was made for PBAY. Coconut PBAYs were significantly higher compared with almond, oat, and soya PBAY and low-fat and very low-fat dairy. For PBAY GS, the one mixed ingredient base and one soya with added coconut fat and two coconut PBAY GS were highest in saturated fat at 7.5–12.8 g per 150 g serving.

***Protein***. Only soya PBD and PBAY varieties had a protein content comparable to dairy with a median of 7.8 g per 250 mL and 6.3 g per 150 g, respectively. For PBAY GS, the three soya and the one mixed ingredient base with soya as the primary base were most comparable to dairy GS yogurts providing 5–8.7 g protein per 150 g serving compared to dairy Greek-style with a median of 6 g per 150 g serving. All other PBAY GS were lower in protein.

***Sugars***. With the exception of rice, all other PBD had median total sugar (intrinsic and extrinsic) values lower than dairy milk (median 1.3 g–8.8 g vs. 12 g per 250 mL serving). A total of 57% of sweetened PBD were low in total sugars, providing no more than 2.5%. Rice PBD had significantly higher total sugar levels with nine out of the seventeen samples containing 33–90% more total sugar compared with dairy. Unsweetened PBD total sugar levels were marginally lower compared with sweetened varieties (median 5.3 g vs. 6.3 g per 250 mL) (Table 6). PBAY provided a median of 1.4 g of total sugars per 150 g serving. Soya PBAY were appreciably lower in total sugars compared to dairy (0.7 g vs. 6.8 g), and other than this, no significant difference in levels was detected between PBAY and dairy. Sweetened PBAY (33%) were higher in total sugars compared with unsweetened PBAY (median 10.1 g vs. 0.8 g total sugars per 150 g serving) (Table 6). No difference in total sugar levels was found between PBAY GS ingredient bases or when compared to dairy.

***Fibre***. Per serving, PBD provided relatively low levels of fibre, with a median of 1 g for PBD, 0.8 g for PBAY, and 1.7 g for PBAY GS. The majority (88%) provided no more than 2 g per serving (8% of the 25 g recommended daily intake of fibre). Dairy milk provided 0-0.8 g per serving and dairy yogurts provided 0–0.2 g per serving. Within our sample, five PBD (soya, almond, pistachio, oat, and oat with hazelnut) and two PBAY (coconut and soya) provided appreciable amounts between 4 g and 5.9 g per serving, which equates to 16–24% of recommended intakes.

***Salt***. With the exception of one oat and one soya drink (1.25 g and 0.9 g of salt, respectively), salt levels in PBD did not exceed 0.5 g per 250 mL, which is only marginally higher than the 0.25 g salt in dairy. Salt levels in PBAY and PBAY GS were also comparable to their dairy counterparts.

### 3.3. Micronutrients in Non-Organic Varieties

Table 7, Table 8 and Table 9 provide an overview of the micronutrient profiles for non-organic PBD, PBAY, and PBAY GS, respectively. Micronutrient values for each product in our analysis can be found in Supplementary Materials Tables S4–S7.

For the micronutrient analysis, the 201 non-organic PBDA products (which are legally permitted to be fortified [67]) were analysed: 154 PBD, 39 PBAY, and all eight PBAY GS. With regard to dairy, all milk and yogurts within our sample were included. However, for dairy Greek-style yogurts, the national food databases of three countries did not provide complete micronutrient information (failing to report on calcium, vitamin B2, and/or vitamin B12), and therefore, only eight of the eleven in our sample with full nutrition information were included.

#### 3.3.1. Fortification

The majority (76%) of non-organic PBDA in our sample were fortified with at least one, 70% with at least two, and 62% with at least three micronutrients. A small number (16) of PBD and two PBAY were fortified with at least five, and two PBD were fortified with the highest number of micronutrients (a total of six). A greater proportion of PBD were fortified (81%) compared to PBAY (62%) and five out of the eight (63%) PBAY GS.

Calcium was the most common fortification (76%), with only one fortified product omitting calcium but including vitamins D, B2, and B12. Sixty-five percent of PBDA were fortified with both calcium and vitamin D; 70% PBD, 46% PBAY, and 63% PBAY GS. Just over half (56%) were fortified with calcium, vitamin D, and vitamin B12; 59% PBD, 44% PBAY, and 63% PBAY GS. Less than half were fortified with vitamin B2, and a few (6%) with vitamin A.

#### 3.3.2. Micronutrients: Calcium, Iodine, and Vitamins D, B2, B12, and A

***Calcium***. The majority of calcium-fortified PBD provided a median of 300 mg of calcium per 250 mL (30% of DRV), comparable to dairy low-fat milk. ‘Other single and mixed base’ PBD provided a significantly lower median of 150 mg of calcium per serving. Sixty-two percent of PBAY were fortified with a median of 180 mg per 150 g (18% DRV), and this level was significantly lower than dairy yogurts, which provided 215 mg per serving. Eight out of the ten (80%) coconut PBAY were not fortified. Sixty-three percent of PBAY GS were fortified with calcium at 180 mg per serving, which is similar to dairy at 189 mg.

***Iodine***. Median iodine levels for the PBDA in our sample were 0 mg, with few fortified; 12% PBD, 8% PBAY, and 1 out of 8 (13%) PBAY GS. Iodine levels in dairy were significantly higher. Oat-based variants were more frequently fortified with iodine compared to the other base ingredients (*p* < 0.05), accounting for 61% of the fortified PBD and all of the fortified PBAY and PBAY GS. The few samples of PBD and PBAY that were fortified provided iodine at levels comparable to dairy, median of 56 mcg and 34 mcg respectively.

***Vitamin D***. Seventy-one percent of PBD, 46% of PBAY, and 63% of PBAY GS were fortified with vitamin D. Far fewer dairy comparisons were fortified with vitamin D: 27% of milk and 18% of yogurts, providing 1.2–2.5 mcg vitamin D per serving. None of the dairy Greek-style yogurts were fortified. Unfortified dairy products provided negligible quantities of naturally occurring vitamin D (0–0.15 mcg per serving). PBD provided a median of 1.88 mcg per serving, which was greater than dairy milk at 0.03 mcg per serving (did not reach significance). PBAY that were fortified (mainly soya and oat variants) provided a median of 1.13 mcg per serving.

***Vitamin B2***. Fifty percent of PBD and 26% of PBAY in our sample were fortified. For the samples that were fortified, levels were comparable to their dairy counterparts; per serving, PBD provided a median of 0.45 mcg vs. 0.53 mcg for dairy milk and PBAY provided 0.32 mg vs. 0.29 mg for dairy yogurts. None of the PBAY GS were fortified.

***Vitamin B12***. Sixty-four percent of PBD, 44% of PBAY, and 63% of PBAY GS were fortified with vitamin B12. Over 60% of soya, oat, almond, and coconut and approximately half of the rice and ‘other single and mixed base’ PBD were fortified. Dairy milk, compared with all PBD, was significantly higher in vitamin B12. PBD that were fortified provided a median vitamin B12 value of 0.95 mg. For PBAY, over half the soya and oat-based varieties were fortified compared with one coconut and almond base. Compared with all PBAY, dairy yogurts were significantly higher in vitamin B12. However, PBAY that were fortified provided higher median levels of 0.57 mcg per serving compared with dairy at 0.45 mcg. PBAY GS provided higher levels of vitamin B12 compared with dairy; however, this did not reach significance.

***Vitamin A***. A very small proportion (6%) of our sample was fortified with vitamin A, and all were PBD. Comparing all PBD median values with dairy milk, the latter provided significantly higher values for vitamin A. However, the handful of PBD that were fortified provided anything from 250 mcg to 1000 mcg per serving, which is greater than dairy milk values of 33–70 mcg.

## 4. Discussion

The results of this study provide an extensive analysis of the macro- and micro-nutrient profiles of European PBDA products. Many studies support the argument that PBDA should be nutritionally comparable to their dairy counterpart, in particular, for protein; calcium; and vitamins A, D, B2, and B12, to reduce any risk of deficiencies [28,41,45,46].

Our study investigated 249 PBD, which is a larger sample size than eight other publications that included 12–148 samples [27,38,39,49,50,51,69,70] but lower than three studies that included 330–641 samples [41,45,71]. We also considered PBAY and PBAY GS on the European market, which were not considered in the majority of previous studies. We identified three studies specifically investigating PBAY in the US and UK [46,47,49]. Our sample of 52 PBAY is comparably smaller than our PBD sample, but it reflects the significantly smaller size of the market [24,32].

This study focused on plain, unflavoured varieties of PBDA and compared them to lower-fat dairy products. This reflects current national FBDG, which discourage flavoured dairy and dairy alternatives. By doing so, we were able to provide important insights into the nutritional content of different dairy products and alternatives, which could inform public health policies and dietary recommendations. Many, but not all, previous studies incorporated both flavoured and sweetened and unsweetened plain PBDA in their analysis without differentiating when comparing to plain dairy [28,39,41,44,46,47,69,70]. The difference in total sugar levels between flavoured and unflavoured varieties can be significant, as highlighted by one study that showed flavoured PBD to provide two to eight times more total sugars compared to unflavoured varieties [69].

### 4.1. Macronutrient and Salt Profile of PBDA Compared to Dairy

Our findings revealed that PBD had a comparable energy content to semi-skimmed milk, with a median of 98 kcal per 250 mL serving, compared to 115 kcal for dairy milk. This finding aligns with the findings of other studies that compared PBD to skimmed, semi-skimmed, and whole milk [27,38,39,41,42,43,44,45,50]. Coconut and almond PBD typically had the lowest energy content, and rice and oat-based varieties provided the most energy. PBAY were significantly higher in energy compared to low-fat dairy, with coconut varieties providing the highest energy content. This finding concurs with a US study that found PBAY (flavoured and plain) to provide 120–170 kcal per 150 g serving [46]. PBAY GS energy values were comparable to dairy in our study.

The protein content and quality of most PBDA, except for soya and, in some cases, pea protein, have been noted as lacking compared with dairy, and the potential risk of protein deficiency has been raised, particularly in vulnerable groups, such as children, the elderly, and individuals with heightened needs [28,38,39,51,71]. Our study found that the majority of non-soya PBDA contained less than 2 g of protein per 100 mL/g, which is significantly lower than dairy products. However, it is important to consider the protein content and quality of PBDA within the context of developed countries’ FBDG, which primarily target non-vulnerable population groups that are generally in good health and consume a varied diet that often exceeds both energy and protein requirements. In addition, most individuals consuming PBDA consume these in conjunction with dairy rather than displacing dairy [26,37,39].

In terms of FBDG, dairy and its alternatives are typically classified outside the protein food group, and overconsumption of protein by the general population is common [72,73]. Furthermore, there are other food sources that are significantly more protein-dense, such as beans, eggs, nuts, and poultry, which can more easily meet protein requirements. Other authors have also presented convincing arguments regarding the limited relevance of focusing solely on the amino acid profile of individual foods, as it does not account for the overall amino acid intake from a mixed diet usually consumed by healthy individuals throughout the day when a nitrogen balance can be achieved [74,75,76,77,78].

When comparing the protein quality of individual PBDA products with their dairy counterparts, standardised methods—Protein Digestibility Corrected Amino Acid Score (PDCAAS) or Digestible Indispensable Amino Acid Score (DIAAS)—indicate that PBDA, with the exception of soya and some pea varieties, have an inferior amino acid profile compared with dairy [28,38,39,42,44,45,51,69]. However, some authors have criticised the significance of solely measuring and comparing the amino acid profiles of individual foods in relation to physiological relevance [73,75,76,77]. It has been well established that achieving nitrogen balance depends on the overall provision of amino acids from various food sources over a 24 h period, rather than the quantity and quality of just one or two specific foods [73,74,75,79]. In developing countries where food sources of protein are limited, the amino acid quality of individual foods can significantly impact an individual’s ability to meet their amino acid requirements throughout the day. However, this is not the case in developed countries, such as in Europe and the United Kingdom, where diets are made up of multiple food protein sources and often exceed protein and energy requirements. Nitrogen balance studies have demonstrated that healthy individuals consuming a varied diet achieve a nitrogen balance regardless of whether the protein source is from animal or plant-based foods [73,74,76,77,79,80]. Thus, while PBDA generally exhibit a lower protein quantity and quality compared to dairy, it is important to consider this in the context of overall dietary intake. In developed countries where diets are diverse and nutritionally adequate, amino acid intakes are not likely to be compromised even when substituting dairy milk with PBDA.

Regarding total fat levels, PBD generally exhibited comparable levels to semi-skimmed milk. However, most PBDA had a lower saturated fat content than semi-skimmed milk, low-fat yogurts, and Greek-style yogurts. The exception was coconut-based PBDA and those with added coconut fat, which provided the highest saturated fat levels (median of 2.8 g for PBD, 10.7 g for PBAY, and 8.8 g for PBAY GS per serving). These findings align with other studies that have reported similar patterns for PBD [27,41,44,46,69,71]. Reducing saturated fat intake is a key recommendation by most dietary guidelines, thus, incorporating PBDA in the diet could help achieve this goal. As coconut drinks and PBAY are gaining popularity, it is crucial to inform consumers about the higher saturated fat content and encourage healthier ingredient choices.

Another criticism of PBDA is their extrinsic sugar content, in contrast to the intrinsic lactose found in dairy. In our analysis, we discovered that only 30% of the plain unflavoured PBDA samples contained added sugars (based on the ingredient list declaration), which is similar to a Swiss analysis that identified 33% of samples as sweetened [38]. This percentage is significantly lower than the findings of a New Zealand analysis, which reported 63% of PBD samples as sweetened [39], and an analysis of European, US, and Australian PBD, as well as US PBAY, which found 84% of PBD and 91% of PBAY samples to be sweetened [27,46]. This difference can be attributed to the inclusion of flavoured PBDA in many of these studies, which are more likely to be sweetened compared with unflavoured plain varieties.

For our investigations, we undertook additional analysis to investigate the differences in total sugar levels between PBDA with and without added sugars. In our samples of PBD, we observed little difference in total sugar levels between sweetened and unsweetened varieties, with 6.3 g and 5.3 g per 250 mL serving, respectively (Table 6). These values were lower than those found in dairy products, which ranged from 11.3 g to 12 g. Similar conclusions were reached by two European studies and one US study [27,45,69]. Rice and oat-based PBDA tended to have higher sugar levels in both sweetened and unsweetened varieties. It is worth noting that for rice and oat drinks, not all the sugars are necessarily added, but significant quantities can result during grain flour processing, which frees sugars from the grain structure. Some oat brands in the market are modifying their production process to remove these sugars, offering zero-sugar versions. Regarding PBAY, the sugar levels in sweetened varieties were higher, with 10.1 g compared with 0.8 g in unsweetened varieties. Another US study that investigated PBAY, including flavoured varieties, reported similar median sugar levels of 10 g (range 7–14 g) [46]. This suggests that for PBDA identifying the total sugar content information, rather than solely relying on “unsweetened” labelling messages, would be more effective in monitoring sugar consumption.

PBDA can provide some fibre, be it at relatively low levels with most achieving no more than 2 g per serving, which would contribute to around 8% of international recommendations for 25 g fibre per day. Only a handful of our PBD samples and two PBAY provided appreciable amounts between 4 g and 5.9 g per serving, which equates to 16–24% of recommended intakes.

Despite some criticism of the addition of salt to PBDA, our analysis identified salt levels to be low at less than 0.3% (with the exception of five samples), with 76% providing no more than 0.3 g salt per serving. These levels are comparable to dairy milk and unlikely to contribute to excess intakes.

### 4.2. Micronutrient Profile of PBDA Compared to Dairy

As PBD and PBAY do not naturally provide significant quantities of micronutrients, many manufacturers voluntarily fortify non-organic varieties. We observed significant disparities in the type and level of fortification, not only among different brands but also among various products within the same brand. These findings align with studies conducted in Europe, the US, and New Zealand [27,38,39,41,42,44,46,49,69].

The situation in Europe is further complicated by the prevalence of “organic” or “bio” varieties, which constituted over one-third of the samples in our study. It is important to note that European regulations prohibit the fortification of organic foods and drinks [67]. This is a crucial factor for governments to take into account when considering the position of PBDA within FBDG, and this distinction needs to be made in the advice to consumers. Fortified PBDA variants that provide essential micronutrients can be positioned as suitable alternatives to dairy products within the FBDG.

Among the non-organic PBD, 76% were fortified with one to six micronutrients, which is consistent with findings from some studies conducted on US and European samples [27,41,50]. Other studies report that a smaller proportion of European PBDA are fortified, ranging from 33% to 50%, possibly due to the inclusion of unfortified organic varieties in their analysis [38,42,51,69,71].

Calcium was the most commonly utilised fortification in PBDA, present in 76% of our sample, usually at levels around 300 mg per serving. This is comparable to dairy products and aligns with investigations into PBDA globally and in Europe. In PBAY, calcium was also frequently added, but with less consistency. Sixty-two percent of our PBAY sample was fortified, and almond-based varieties tended to be fortified at higher levels compared with other ingredient bases (median 216 mg vs. 180 mg). This is similar to a US study, which reported 47% of PBAY to be fortified [46]. The inclusion of dairy within FBDG is primarily for the provision of readily available calcium. Thus, the addition of calcium to PBDA would be beneficial when considering their inclusion in FBDG. However, it is also important to better educate consumers on the ubiquitous presence of calcium in the diet and how they can incorporate other rich sources.

Several authors suggest that the bioavailability of calcium carbonate and tri-calcium phosphate, the main fortificants used in PBDA, is lower than that of dairy calcium [38,39,43,44,45,51,69,70]. It is, however, interesting when interpreting this difference in the context of real dietary intakes. For example, one study comparing cow’s milk and soya drinks fortified with either calcium carbonate or tri-calcium phosphate found calcium bioavailability to be 21.7%, 21.1%, and 18.1%, respectively [78,81]. Thus, for a 250 mL serving providing 300 mg calcium, the difference in bioavailable calcium between dairy and PBDs will be 10–11 mg in absolute value. It remains unclear whether this marginal difference is physiologically significant.

In several European countries, dairy products have become a significant source of iodine, largely influenced by farming practices and the use of iodine-fortified cattle feed [82]. In our sample from 11 European countries, low-fat dairy milk had a median iodine level of 30 mcg (20% of DRV) per serving, with a significant range of 22–75 mcg. Dairy yogurts in our sample had a lower median iodine level of 22.5 mcg per serving, with a range of 5–80 mcg per serving. In contrast, only 13% of our non-organic PBD and PBAY samples and one PBAY GS were fortified with iodine, at levels ranging from 33 to 90 mcg per serving. Limited information is available regarding the iodine fortification of PBDA, but studies have indicated that they are generally not fortified [38,45,51,71]. In countries where dairy is a significant source of iodine, and fish (especially white and shellfish) and/or seaweed intake is low, fortifying PBDA with iodine may help with the suboptimal iodine status of populations in many European countries [82,83,84]. However, the use of iodised salt and fortification has faced resistance in certain European countries due to regulatory and practical concerns, as well as worries regarding potential toxicity. Addressing these concerns is necessary for the successful implementation of iodine fortification in PBDA across all European countries [85,86].

Dairy in Europe is perceived as a significant source of vitamin D, often based on US publications, where the fortification of dairy milk with vitamin D is mandatory in most states [38,41,42,69]. However, this is not the case with all European countries [87,88]. Europe typically does not fortify dairy products, as evidenced by our study. The vitamin D content of 11 dairy milk samples, using national food databases, ranged from 0 to 2.5 mcg per serving [55,56,57,58,59,60,61,62,63,64,65]. Among our samples, only dairy milk from Finland, Sweden, and Belgium was fortified at levels of 1.5–2.5 mcg per serving. The national food databases of the UK, Switzerland, Spain, Denmark, and the Netherlands showed little to no vitamin D content in milk (0–0.23 mcg per 250 mL serving). This finding is consistent with other publications that cite the standard and frequent fortification of dairy in Sweden, Norway, Finland, Belgium, and Spain, with levels ranging from 0.38 mcg to over 1 mcg per 100 g (0.95–2.5 mcg per serving) [87].

In our analysis of non-organic PBDA, we found that only 46% of the samples were fortified with vitamin D. PBD had a higher fortification rate at 71% compared with PBAY (61%). This corresponds to other studies that have identified fewer than 50% of European PBDA products being fortified with vitamin D [38,41,44,46,51,71]. When fortification was present, the levels in PBD (1.88 mcg per serving) and PBAY (1.35 mcg per serving) were similar to their fortified dairy counterparts. The addition of vitamin D in PBDA can support calcium bioavailability; however, with regard to considering PBDA’s suitability as a dairy alternative, this will depend on a country’s practice for dairy fortification.

Vitamin B12 fortification was more common in our sample of non-organic PBD (64%) and PBAY GS (63%) compared with PBAY (44% fortified), providing levels similar to their dairy counterparts. Other studies have observed infrequent vitamin B12 fortification in European PBDA, ranging from 21% to 40% [27,38,41,46,71]. In the US, a significantly higher proportion of single-serving PBD were fortified compared to multi-serving packed PBD, with fortification rates of 65% and 34–47%, respectively. Vitamin B12 can be obtained from various animal food sources within the diet. Considering the current evidence, it is apparent that consumers of PBDA still consume other dairy products [25,36,37]. Additionally, it is worth noting that although the popularity of vegan diets is increasing, this still represents a relatively small proportion (2–3.4%) of the European population [89,90]; thus, for the majority, vitamin B12 requirements can easily be achieved by consuming other dairy, meat, and animal foods. On the other hand, vegan groups are more likely to consume PBDA on a regular basis, and with few plant foods fortified with vitamin B12, the fortification of PBDA would be beneficial.

Dairy is a natural source of vitamin B2 with 250 mL of low-fat milk meeting 23–28% of the recommended DRV, and a 150 g serving of low-fat yogurt and Greek-style yogurt meeting 18% of the DRV. In our sample, 50% of PBD and 26% of PBAY were fortified with vitamin B2, reaching levels similar to or surpassing those found in dairy products. This finding is consistent with other studies reporting vitamin B2 fortification rates of 0–30% in PBD [38,41,44,71]. Additionally, there were variations in fortification levels across different ingredient bases. In our sample, none of the eight PBAY GS products were found to be fortified with vitamin B2. Considering that dairy and meat are primary sources of vitamin B2 in the European diet, it would be advantageous to fortify PBDA to mitigate the risk of inadequate intakes when individuals decide to replace dairy with PBDA.

Within our dairy sample, significant amounts of vitamin A were only found in Greek-style yogurt. Vitamin A fortification was uncommon in our PBDA samples, with only 13 PBD products, primarily those sold in Spain, containing this nutrient.

### 4.3. Strengths and Limitations

Our study had several strengths that contribute to its robustness. Firstly, our sample size was significantly greater than a number of previous publications. Secondly, we have provided a comprehensive review of leading PBDA brands across European rather than focusing on one specific country. Finally, excluding organic variants from our micronutrient analysis provides a more realistic and practical comparison of the PBDA in context of inclusion within FBDG.

However, it is important to acknowledge some limitations of our study. Our nutritional information is based on PBDA producers’ food-labelling declarations available online, and for dairy, from national food databases. Thus, where nutrient values were not present, we automatically allocated a zero value. Another limitation is that we did not collect full ingredient information for the products. Having this information may have helped us better understand the sources of vitamins and minerals, as well as the types of sugars added to the product.

### 4.4. Further Research

Our study’s aim and objectives focused solely on comparing the macro and micronutrient content of PBDA with those of dairy. These nutritional levels are commonly cited as a concern. With the increasing popularity of PBDA, future research could further investigate other ingredients present within PBDA and the degree of processing, as well as the role of PBDA with regard to health outcomes, environmental impacts, and socio–economic factors such as cost.

## 5. Conclusions

In conclusion, transitioning towards dietary patterns centered around healthful plant foods and reducing reliance on meat and dairy agriculture is crucial for human and planetary health in developed countries. Our study highlights that PBDA can be part of this transition and fit well within sustainable dietary patterns.

Our comprehensive analysis demonstrates that PBDA can be a healthy choice within a diverse diet and in the context of public health. They have comparable energy and sugar levels to dairy products and serve as excellent low-saturated fat alternatives, with the exception of coconut-based and added coconut fat varieties. Soya PBDA provide protein levels and quality on par with dairy.

To address current concerns about the nutritional quality of PBDA, improved fortification with key European dairy micronutrients would be beneficial. The current regulatory prohibition on the fortification of organic foods and drinks makes it impossible to fortify organic PBDA. However, for non-organic PBDA, gaps in the fortification could be better addressed, and our analysis demonstrates that when micronutrients are added to PBDA, they are often at levels comparable to European dairy. We identified 24%, 40%, and 57% of non-organic PBDA in our sample not fortified with calcium, vitamin B12, and vitamin B2, respectively. Improved fortification is needed across all categories to rectify the existing imbalance between PBD and PBAY fortification. Furthermore, in countries (not all) where dairy plays a significant role in vitamin D and iodine provision, optimising the fortification of these micronutrients in PBDA will be particularly helpful, as there are few other food sources.

In summary, fortified PBDA can help shift consumers towards more sustainable eating patterns, and their macronutrient profile, except for coconut varieties, is conducive to improved health outcomes. As PBDA continue to grow in popularity, there is a need to have consistency in micronutrient fortification and help consumers incorporate them in the context of a healthful and varied diet.

## Figures and Tables

**Table 1:** Base ingredient/s for “other” plant-based drinks.

Single-Base	No.	Mixed-Base	No.
Pea protein	3	Soya and oat	6
Spelt	2	Coconut and rice	4
Cashew	1	Oat and almond	3
Hemp	1	Almond, corn, and oat	1
Pistachio	1	Almond and hazelnut	1
		Coconut and soya	1
		Hemp and hazelnut	1
		Mixed nuts	1
		Oat and barley	1
		Oat and hazelnut	1
		Oat and hemp	1
		Soya and almond	1
		Soya, date, and almond	1
		Soya and rice	1
		Soya, rice, oat, and almond	1
Total	8	Total	25

**Table 2:** Base ingredients for mixed-base plant-based alternatives to yogurts.

Mixed-Base PBAY	No.
Soya and coconut	3
Coconut and pea protein	2
Almond, pea protein, and hazelnut	1
Almond, pea protein, and pistachio	1
Coconut and almond	1
Oat, coconut, and hazelnut	1
Oat and coconut	1
Total	10

**Table 3:** Median (range) macronutrient values of 249 plant-based drinks (PBD) and dairy low-fat (LF) milk per 250 mL single serving.

Base	Soya	Oat	Almond	Coconut	Rice	Other Single and Mixed	ALL PBD	Dairy LF Milk	*p* Value for Differences between Bases	*p* Value between All PBD and Dairy
n	57	69	50	23	17	33	249	11		
kJ	405 (233–645) ^a,b^	483 (265–743) ^a^	250 (125–630) ^b,c,g^	213 (125–563) ^a,d,e^	553 (228–723) ^b,f^	450 (153–610) ^c,e^	413 (125–743)	488 (473–503) ^d,g^	<0.001	0.011
kcal	98 (55–155) ^a–e^	115 (63–178) ^a,f,g^	60 (30–150) ^b,f,h-j^	50 (30–135) ^c,g,k–m^	133 (55–170) ^d,h,k^	108 (38–145) ^i,l^	98 (30–178)	115 (113–120) ^e,j,m^	<0.001	0.012
Fat g	4.5 (2.5–11) ^a–d^	3.8 (1.3–8.8) ^a,e^	3.3 (2.3–10) ^b,f^	3.3 (1–10)	2.8 (1.3–3.5) ^c,e,f-h^	4.3 (2.3–9.5) ^g^	3.8 (1–11)	3.8 (3–4.3) ^d,h^	<0.001	0.935
Saturated fat g	0.8 (0.3–1.5) ^a,b^	0.5 (0.3–2.3) ^a,c^	0.3 (0.3–1) ^a,d^	2.8 (0.3–7.8) ^a,e,f^	0.5 (0.3–1) ^b,e^	0.5 (0–3.5) ^d,f^	0.5 (0–7.8)	2.5 (2–2.8) ^b,c,d^	<0.001	<0.001
Carbohydrate g	5.8 (0–40) ^a,b^	16.8 (8.3–24) ^a,c,d^	2.9 (0–26.3) ^d,e,f^	5.3 (0–26) ^c,g^	25 (9.3–35) ^a,e,g^	10 (0.3–27.5) ^a,f,g^	8 (0–40)	12 (11.3–12.3) ^b,c,e^	<0.001	0.252
Sugars g	4.3 (0–8.5) ^a-c^	8.8 (0–16.3) ^a,d,e^	1.3 (0–12) ^e,f,g^	4.8 (0–11.3) ^d,h,i^	16 (0–22.8) ^b,d,f^	8.3 (0–20) ^c,g,h^	6 (0–22.8)	12 (11.3–12.3) ^a,e,i^	<0.001	<0.001
Fibre g	1.3 (0–4.0) ^a,b^	1.8 (0–4.3) ^c,d^	0.8 (0–4.5) ^c^	0.8 (0–2.3) ^e^	0.3 (0–1.3) ^a,d^	0.3 (0–4.3)	1 (0–4.5)	0 (0–0.8) ^b,c,e^	<0.001	<0.001
Protein g	7.8 (5–9.8) ^a,b^	2 (0–4) ^a,c^	1.3 (0–3.8) ^a,d^	1.3 (0.3–4.3) ^b,e^	0.5 (0.3–1.3) ^a,f,g^	2.8 (0–8.5) ^b,d,f^	2 (0–9.8)	8.5 (7.5–10) ^c,d,e,g^	<0.001	<0.001
Salt g	0.25 (0.03–0.90)	0.25 (0.08–1.25) ^a^	0.3 (0–0.5)	0.25 (0.03–0.33)	0.23 (0.13–0.5) ^b^	0.33 (0–0.48) ^a,b^	0.25 (0–1.25)	0.25 (0.2–0.3)	0.002	0.828

Comparisons between all PBD and dairy low-fat (semi-skimmed) milk were carried out using the Mann–Whitney U test for independent samples. Comparisons between the different PBD ingredient bases and dairy milk were made using the Kruskal–Wallis test followed by the Dwass–Steel–Critchlow–Fligner test for multiple comparison analysis. The significance level was set at *p* < 0.05. Superscript letters (a–m) in the same row indicate significant differences (*p* < 0.05) among base types for the related nutrient.

**Table 4:** Median (range) macronutrient values of 52 plant-based alternatives to yogurt (PBAY) and dairy low-fat (LF) and very low-fat (VLF) yogurts per 150 g single serving.

Base	Soya	Oat	Almond	Coconut	Mixed	ALL PBAY	Dairy LF	Dairy VLF	ALL DAIRY	*p* Value for Differences between Bases	*p* Value between All PBAY and Dairy
n	20	7	4	11	10	52	7	10	17		
kJ	297 (221–554) ^a,b^	519 (245–525)	508 (327–741)	605 (390–1089) ^a,c,d^	575 (312–789) ^b,e,f^	462 (221–1089)	317 (287–365) ^c,e^	248 (213–345) ^d,e^	287 (213–365)	<0.001	<0.001
kcal	71 (62–132) ^a,b^	123 (59–126)	122 (78–179)	146 (93–260) ^a,d,e^	138 (75–189) ^b,c^	110 (59–260)	75 (69–86) ^c,d^	59 (51–81) ^b,e^	69 (51–86)	<0.001	<0.001
Fat g	3.5 (2.4–5.1) ^a–c^	4.5 (1.4–6.8) ^d^	7.4 (5–14.3) ^a^	11.6 (6–25.5) ^b,d,e^	6.8 (3.5–11.6) ^c^	5 (1.4–25.5)	2.3 (1.5–2.4) ^c,e^	0.2 (0.2–0.8) ^c,d^	0.5 (0.2–2.4)	<0.001	<0.001
Saturated fat g	0.6 (0.3–0.9) ^a,b^	0.5 (0.2–5.6) ^c^	0.6 (0.5–1.2) ^d^	10.7 (5–22.5) ^a,c^	5.6 (0.6–8.4) ^b,e^	0.7 (0.2–22.5)	1.5 (1.1–1.5) ^a^	0.2 (0–0.5) ^a,d,e^	0.2 (0–1.5)	<0.001	0.003
Carbohydrates g	1.1 (0–18) ^a–d^	16.5 (5.9–20.9) ^a^	8.5 (7.5–9.8)	8.1 (5.6–16.8) ^b^	18.8 (0.9–25.1)^c^	7.5 (0–25.1)	6.8 (3.9–11.7)	7.4 (6–12.3) ^d^	7.2 (3.9–12.3)	<0.001	0.900
Sugars g	0.7 (0–15) ^a,b^	6.6 (0.6–8.7)	1.7 (0.6–5.3)	1.2 (0–10.8)	8.0 (0–18.8)	1.4 (0–18.8)	6.5 (4.1–11.3) ^a^	7.3 (4.8–11.9) ^b^	6.8 (4.1–11.9)	0.002	0.001
Fibre g	0.8 (0–4.7) ^a^	0.6 (0–1.5) ^b^	2.0 (1.5–3.0)^c^	0.0 (0–5.9)	0.4 (0–1.5)	0.8 (0–5.9)	0 (0–0.2)	0 (0–0) ^a,b,c^	0 (0–0.2)	<0.001	<0.001
Protein g	6.3 (5–9) ^a–d^	2 (1.1–2.3) ^a,e,f^	3.1 (0.8–4.1) ^b^	1.2 (0.6–2.3) ^c,g,h^	3.1 (0.8–6.3) ^d,i^	3.8 (0.6–9)	6.2 (5.4–7.2) ^e,g^	6.2 (4.7–8.1) ^f,h,i^	6.2 (4.7–8.1)	<0.001	<0.001
Salt g	0.15 (0.06–0.38)	0.15 (0.11–0.2)	0.11 (0.05–0.15)	0.11 (0.05–0.6)	0.13 (0.02–0.29)	0.15 (0.02–0.6)	0.17 (0.15–0.24)	0.2 (0.14–0.3)	0.2 (0.14–0.3)	0.062	0.004

Comparisons between all PBAY and all dairy yogurts (very low-fat and low-fat combined) were carried out using the Mann–Whitney U test for independent samples. Comparisons between the different PBAY ingredient bases and dairy low-fat and very low-fat yogurts were made using the Kruskal–Wallis test followed by the Dwass–Steel–Critchlow–Fligner test for multiple comparison analysis. The significance level was set at *p* < 0.05. Superscript letters (a–i) in the same row indicate significant differences (*p* < 0.05) among base types for the related nutrient.

**Table 5:** Median (range) macronutrient values of eight plant-based alternatives to Greek-style yogurts (PBAY GS) and 11 dairy Greek-style yogurts per 150 g single serving.

Base	Soya	Oat	Coconut	Mixed	ALL PBAY GS	Dairy Greek-style	*p* Value for Differences between Bases	*p* Value between All PBAY GS and Dairy
n	3	2	2	1	8	11		
kJ	425 (378–750)	848 (791–905)	680 (656–704)	782 (782–782)	727 (378–905)	693 (405–827)	0.133	0.657
kcal	102 (90–180)	203 (189–218)	164 (159–170)	189 (189–189)	175 (90–218)	168 (96–200)	0.137	0.620
Fat g	5 (5–15)	13.7 (12.5–15)	13.4 (12.5–14.4)	15 (15–15)	13.4 (5–15)	13.7 (0.3–15.3)	0.773	0.868
Saturated fat g	0.9 (0.9–8.7)	1.2 (1.2–1.2)	12.2 (11.7–12.8)	7.5 (7.5–7.5)	4.4 (0.9–12.8)	8.9 (0–10.2)	0.065	0.482
Carbohydrates g	3.9 (0–7.1)	16 (15–17)	8.8 (8.6–9)	6.6 (6.6–6.6)	7.8 (0–17)	6.3 (4.4–9.0)	0.049	0.283
Sugars g	3.6 (0–3.8)	5 (3.8–6.2)	0.8 (0.8–0.9)	0.9 (0.9–0.9)	2.3 (0–6.2)	5.7 (4.4–6.8)	0.075	0.020
Fibre g	2 (0–2.3)	2.2 (1.4–3) ^a^	1.5 (0.2–2.9) ^b^	0 (0–0)	1.7 (0–3)	0.0 (0–0.2) ^a,b^	0.014	0.003
Protein g	8.7 (5–8.7)	3 (1.1–5)	1.4 (1.2–1.7)	6.8 (6.8–6.8)	5 (1.1–8.7)	6.0 (4.8–16.2)	0.092	0.148
Salt g	0.5 (0.11–0.54)	0.14 (0.11–0.17)	0.24 (0.15–0.33)	0.21 (0.21–0.21)	0.19 (0.11–0.54)	0.17 (0.14–0.26)	0.559	0.503

Comparisons between all PBAY GS and all dairy Greek-style yogurts were carried out using the Mann–Whitney U test for independent samples. Comparisons between the different PBAY ingredient bases and dairy Greek-style yogurts were made using the Kruskal–Wallis test followed by the Dwass–Steel–Critchlow–Fligner test for multiple comparison analysis. The significance level was set at *p* < 0.05. Superscript letters (a and b) in the same row indicate significant differences (*p* < 0.05) among base types for the related nutrient.

**Table 6:** Comparing median total sugars per serving between plant-based dairy alternatives with and without added sugars.

	Total Sugars Median (Range), g		
Serving	250 mL PBD	150 g PBAY	150 g PBAY GS
With added sugars	*n* = 74 (30%)	*n* = 17 (33%)	*n* = 2 (25%)
	6.3 (3.0–17.3)	10.1 (0.6–18.8)	3.8 (3.8–3.8)
Without added sugars	*n* = 175 (70%)	*n* = 35 (67%)	*n* = 6 (75%)
	5.3 (0–22.8)	0.8 (0–8.7)	0.9 (0–6.2)

PBD = plant-based drinks, PBAY = plant-based alternative to yogurts, PBAY GS = plant-based alternatives to Greek-style yogurt.

**Table 7:** Micronutrient content of 154 non-organic plant-based drinks (PBD) and dairy low-fat (LF) milk. Micronutrient value as median (range) per 250 mL single serving and contribution (%) to EFSA adult Dietary Reference Values (DRV).

	Base	Soya	Oat	Almond	Coconut	Rice	Other Single and Mixed	All PBD	Dairy Low-Fat Milk	*p* Value for Differences between Bases	*p* Value between All PBAY GS and Dairy
	*n*	35	49	33	14	6	17	154	11		
Calcium	No. fortified	28	42	28	12	4	9	123	NA		
	Median (range) mg	300 (0–360)	300 (0–320)	300 (0–463)	300 (0–425)	300 (0–300)	150 (0–300) ^a^	300 (0–463)	300 (285–388) ^a^	0.006	0.004
	%DRV	30% (0–36%)	30% (0–32%)	30% (0–46%)	30% (0–43%)	30% (0–30%)	15% (0–30%)	30% (0–46%)	30% (29–39%)		
Iodine	No. fortified	2	11	2	3	0	0	18	NA		
	Median (range) mcg	0 (0–56) ^a^	0 (0–90) ^b^	0 (0–56) ^c^	0 (0–56) ^d^	0 ^e^	0 ^f^	0 (0–90)	30 (22–75) ^a–f^	<0.001	<0.001
	%DRV	0% (0–38%)	0% (0–60%)	0% (0–38%)	0% (0–38%)	0%	0%	0% (0–60%)	20% (14–50%)		
Vitamin D	No. fortified	23	41	22	11	4	8	109	3		
	Median (range) mcg	1.88 (0–3.75)	1.88 (0–3.75) ^a^	1.88 (0–3.75)	1.88 (0–3)	1.88 (0–3.75)	0 (0–3.75) ^a^	1.88 (0–3.75)	0.03 (0–2.5)	0.009	0.064
	%DRV	13% (0–25%)	13% (0–25%)	13% (0–25%)	13% (0–20%)	13% (0–25%)	0% (0–25%)	13% (0–25%)	0% (0–17%)		
Vitamin B2	No. fortified	21	28	17	5	2	4	77	NA		
	Median (range) mg	0.53 (0–0.53)	0.53 (0–0.53)	0.50 (0–0.53)	0 (0–1.25)	0 (0–0.95)	0 (0–0.53)	0.25 (0–1.25)	0.45 (0.38–0.6)	0.293	0.603
	%DRV	33% (0–33%)	33% (0–33%)	33% (0–33%)	0% (0–78%)	0% (0–59%)	0% (0–33%)	16% (0–78%)	28% (23–38%)		
Vitamin B12	No. fortified	21	36	21	9	3	8	98	NA		
	Median (range) mcg	0.95 (0–0.95) ^a^	0.95 (0–1.73) ^b^	0.95 (0–0.95) ^c^	0.73 (0–0.95) ^d^	0.48 (0–0.95)	0 (0–0.95) ^e^	0.95 (0–1.73)	1.1 (0.63–2.25) ^a–e^	<0.001	<0.001
	%DRV	24% (0–24%)	24% (0–43%)	24% (0–24%)	18% (0–24%)	24% (0–24%)	0% (0–24%)	24% (0–43%)	28% (16–56%)		
Vitamin A	No. fortified	5	1	5	2	0	0	13	11		
	Median (range) mcg	0 (0–300) ^a^	0 (0–300) ^b^	0 (0–1000) ^c^	0 (0–250) ^d^	0 ^e^	0 ^f^	0 (0–1000)	43 (33–70) ^a–f^	<0.001	<0.001
	%DRV	0% (0–40%)	0% (0–40%)	0% (0–133%)	0% (0–33%)	0%	0%	40% (0–133%)	6% (4–9%)		

NA = micronutrients naturally occurring (not fortified). Comparisons between all non-organic PBD and all dairy low-fat (semi-skimmed) dairy milk were carried out using the Mann–Whitney U test for independent samples. Comparisons between the different PBD ingredient bases and dairy milk were made using the Kruskal–Wallis test followed by the Dwass–Steel–Critchlow–Fligner test for multiple comparison analysis. The significance level was set at *p* < 0.05. Superscript letters (a–f) in the same row indicate significant differences (*p* < 0.05) among base types for the related nutrient.

**Table 8:** Micronutrient content of 39 non-organic plant-based alternatives to yogurts (PBAY) and dairy yogurts. Micronutrient values as median (range) per 150 g single serving and contribution (%) towards EFSA adult Dietary Reference Values (DRV).

	Base	Soya	Oat	Almond	Coconut	Mixed	All PBAY	Dairy LF	Dairy VLF	All Dairy	*p* Value for Differences between Bases	*p* Value between All PBAY GS and All Dairy
	*n*	13	6	3	10	7	39	7	10	17		
Calcium	No. fortified	10	5	2	2	5	24	NA	NA	NA		
	Median (range)	180 (0–180) ^a,b^	180 (0–180) ^c^	216 (0–225)	0 (0–192) ^d,e^	180 (0–180) ^f^	180 (0–225)	209 (171–243) ^a,d^	219 (180–240) ^b,c,e,f^	215 (171–243)	<0.001	<0.001
	%DRV	18% (0–18%)	18% (0–18%)	22% (0–23%)	0% (0–19%)	18% (0–18%)	18% (0–23%)	21% (17–24%)	22% (18–24%)	21% (17–24%)		
Iodine	No. fortified	0	3	0	0	0	3	NA	NA	NA		
	Median (range)	0 ^a,b^	17 (0–34)	0	0 ^c,d^	0 ^e,f^	0 (0–34)	23 (5–51) ^a,c,e^	18 (0–80) ^b,d,f^	23 (0–80)	<0.001	<0.001
	%DRV	0%	11% (0–23%)	0%	0%	0%	0% (0–23%)	15% (4–34%)	12% (0–53%)	15% (0–53%)		
Vitamin D	No. fortified	8	5	1	1	3	18	2	1	3		
	Median (range)	1.13 (0–2.25)	1.58 (0–2.25) ^a^	0 (0–1.35)	0 (0–1.13) ^a^	0 (0–2.25)	0 (0–2.25)	0.08 (0–1.2)	0 (0–1.5)	0.05 (0–1.5)	0.055	0.532
	%DRV	8% (0–15%)	11% (0–15%)	0% (0–9%)	0% (0–8%)	0% (0–15%)	0% (0–15%)	1% (0–8%)	0% (0–10%)	0% (0–10%)		
Vitamin B2	No. fortified	3	3	1	0	3	10	NA	NA	NA		
	Median (range)	0 (0–0.32) ^a^	0.16 (0–0.32)	0 (0–0.38)	0 (0–0) ^b,c^	0 (0–0.32)	0 (0–0.38)	0.33 (0.26–0.39) ^a,b^	0.28 (0–0.39) ^c^	0.29 (0–0.39)	<0.001	<0.001
	%DRV	0% (0–20%)	10% (0–20%)	0% (0–23%)	0% (0–0%)	0% (0–20%)	0% (0–23%)	21% (16–24%)	17% (0–24%)	18% (0–24%)		
Vitamin B12	No. fortified	7	5	1	1	3	17	NA	NA	NA		
	Median (range)	0.57 (0–0.9)	0.57 (0–0.6)	0 (0–0.68)	0 (0–0.57) ^a,b^	0 (0–0.57)	0 (0–0.9)	0.5 (0.3–0.6) ^a^	0.44 (0.39–0.66) ^b^	0.45 (0.3–0.66)	0.016	0.008
	%DRV	14% (0–23%)	14% (0–15%)	0% (0–17%)	0% (0–14%)	0% (0–14%)	0% (0–23%)	12% (8–15%)	11% (10–17%)	11% (8–17%)		
Vitamin A	No. fortified	0	0	0	0	0	0	NA	NA	NA		
	Median (range) mcg	0 ^a^	0 ^b,e^	0	0 ^c,f^	0 ^d,g^	0	24 (9–42) ^a–d^	2 (0–14) ^a,e–g^	9 (0–42)	<0.001	<0.001
	%DRV	0%	0%	0%	0%	0%	0%	3% (1–6%)	0% (0–2%)	1% (0–6%)		

NA = micronutrients naturally occurring (not fortified). Comparisons between all non-organic PBAY and dairy yogurts were carried out using the Mann–Whitney U test for independent samples. Comparisons between the different ingredient bases and dairy LF and VLF yogurts were made using the Kruskal–Wallis test followed by the Dwass–Steel–Critchlow–Fligner test for multiple comparison analysis. The significance level was set at *p* < 0.05. Superscript letters (a–g) in the same row indicate significant differences (*p* < 0.05) among base types for the related nutrient.

**Table 9:** Micronutrient content of eight non-organic plant-based alternatives to Greek-style yogurts (PBAY GS) and eight dairy Greek-style (GS) yogurts. Micronutrient values as median (range) per 150 g single serving and contribution (%) towards EFSA adult Dietary Reference Values (DRV).

	Base	Soya	Oat	Coconut	Other	All PBAY GS	Dairy GS	*p* Value for Differences between Bases	*p* Value between All PBAY GS and Dairy
	n	3	2	2	1	8	8 ^1^		
Calcium	No. Fortified	2	2	1	0	5	NA		
	Median (range) mg	180 (0–180)	180 (180–180)	120 (0–240)	0	180 (0–240)	187 (161–219)	0.411	0.093
	%DRV	18% (0–18%)	18% (18–18%)	12% (0–24%)	0%	18% (0–24%)	19% (16–22%)		
Iodine	No. Fortified	0	1	0	0	1	NA		
	Median (range) mcg	0	17 (0–34)	0	0	0 (0–34)	22 (16–59)	0.046	0.004
	%DRV	0%	11% (0–23%)	0%	0%	0% (0–23%)	15% (11–39%)		
Vitamin D	No. Fortified	2	2	1	0	5	0		
	Median (range) mcg	1.13 (0–1.13)	1.39 (1.13–1.65)	0.56 (0–1.13)	0	1.13 (0–1.65)	0.15 (0–0.15)	0.184	0.013
	%DRV	8% (0–8%)	9% (8–11%)	4% (0–8%)	0%	8% (0–11%)	1% (0–1%)		
Vitamin B2	No. Fortified	0	0	0	0	0	NA		
	Median (range) mg	0	0	0	0	0	0.27 (0.2–0.42)	0.011	<0.001
	%DRV	0%	0%	0%	0%	0%	17% (13–26%)		
Vitamin B12	No. Fortified	2	2	1	0	5	NA		
	Median (range) mcg	0.57 (0–0.57)	0.57 (0.57–0.57)	0.29 (0–0.57)	0	0.57 (0–0.57)	0.36 (0.3–1.05)	0.400	0.426
	%DRV	14% (0–14%)	14% (14–14%)	7% (0–14%)	0%	14% (0–14%)	9% (8–26%)		
Vitamin A	No. Fortified	0	0	0	0	0	NA		
	Median (range) mcg	0	0	0	0	0	133 (35–173)	0.012	<0.001
	%DRV	0%	0%	0%	0%	0%	18% (5–23%)		

^1^ The Norwegian, Swiss, and French national food databases did not provide calcium and/or vitamin B12 or B2 values for their Greek-style dairy yogurts, and therefore, these three samples were excluded from the analysis. NA = micronutrients naturally occurring (not fortified). Comparisons between all eight non-organic PBAY GS and dairy Greek-style yogurts were carried out using the Student *t*-test for independent samples. Comparisons between the different PBAY GS ingredient bases and dairy Greek-style yogurts were made using the Kruskal–Wallis test followed by the Dwass–Steel–Critchlow–Fligner test for multiple comparison analysis. The significance level was set at *p* < 0.05. No significant difference was found between the different ingredient bases.

## Supplementary Materials

The following supporting information can be downloaded at: https://www.mdpi.com/article/10.3390/nu15153415/s1, Table S1: European Food Safety Authority Adult (≥18 years) Dietary Reference Values (DRV) for micronutrients used in our analysis; Table S2: 27 brands of plant-based dairy alternatives included in our analysis; Table S3: private (retail) brand labels of plant-based drinks and alternatives to yogurt across seven European countries included in our analysis; Table S4: dairy milk and yogurt macro and micronutrient values per 100 mL/g as declared by 11 European National Food Databases; Table S5: plant-based alternatives to drinks’ macro and micronutrient values as declared by manufacturers/retailers per 100 mL; Table S6: plant-based alternatives to yogurts’ macro and micronutrient values as declared by manufacturers/retailers per 100 g; Table S7: plant-based alternatives to Greek-style yogurts’ macro and micronutrient values as declared by manufacturers/retailers per 100 g.
