# Supplementary material for: A Comprehensive Analysis of the Nutritional Composition of Plant-Based Drinks and Yogurt Alternatives in Europe
Source: Nutrients. 2023 Jul 31;15(15):3415. doi: 10.3390/nu15153415 (PMC10421432; doi:10.3390/nu15153415)
Supplement: Supplementary file 1 [file nutrients-15-03415-s001.zip › EU DRVs and PBDA brands Suppl tables S1-S3.pdf]

Supplementary Material

Tables S1–S3

Table S1. European Food Safety Authority Adult (≥18 years) Dietary Reference Values (DRV) for micronutrients used in our analysis.

Table S2. 27 brands of plant-based dairy alternatives included in our analysis

Table S3. Private (retail) brand labels of plant-based drinks and alternatives to yogurt across seven European countries included in our analysis

Supplementary Material. Table S1. European Food Safety Authority Adult (≥18 years) Dietary Reference Values (DRV) for micronutrients used in our analysis.

Micronutrient	DRV	PRI or AI value
Calcium mg	1000	PRI
Iodine mcg	150	AI
Vit D mcg	15	AI
Vit B2 mg	1.6	PRI
Vit B12 mcg	4	AI
Vit A mcg	750	PRI

PRI = population reference intake; reflects the intake of a nutrient that is likely to meet the needs of almost all healthy people in a population. AI=adequate intake; used when PRI is not available and reflects the average nutrient level, based on observations or experiments, that is assumed to be adequate for the population's needs.

European Food Safety Authority Dietary Reference Values for the EU. DRV Finder. Online interactive tool. https://multimedia.efsa.europa.eu/drvs/index.htm

Supplementary Material. Table S2. 27 brands of plant-based dairy alternatives included in our analysis

Brand	Country
AdeZ - Coca cola	Czech Republic
	Europe
	France
	Hungary
	Italy
	Spain
	UK
Almendrola	Spain
Alnatura	Germany
Alpro	Austria
	Belgium
	Bulgaria
	Croatia
	Czech Republic
	Denmark
	Estonia
	Finland
	France
	Germany
	Greece
	Hungary
	Ireland
	Italy
	Latvia
	Lithuania
	Norway
	Poland
	Portugal
	Republic of Belarus
	Romania
	Russian Federation
	Slovakia
	Slovenia
	Spain
	Sweden
	Switzerland
	The Netherlands
	Turkey
	UK
	Ukraine
Andros	France
	Germany
Bakoma	Poland
Berief	Germany
BJORG	France
	UK
Blue Diamond - Amond Breeze	Finland
	Germany
	Spain
	UK
Califia	Ireland
	UK
Granarolo	Italy
Jord	Denmark
	Sweden
	UK
Joya	Austria
Karpos/Carpos	Greece
Koko	Ireland
	UK
Naturli	Denmark

Supplementary Material. Table S2 continued

Brand	Country
Oatly	Austria
	Belgium
	Croatia
	Czech Republic
	Denmark
	Estonia
	Finland
	France
	Germany
	Hungary
	Iceland
	Ireland
	Latvia
	Lithuania
	Luxembourg
	Norway
	Poland
	Portugal
	Slovenia
	Spain
	Sweden
	Switzerland
	The Netherlands
	UK
Planti	Sweden
Provamel	Belgium
	France
	Germany
	Ireland
	Italy
	Portugal
	Spain
	Switzerland
	The Netherlands
Rude Health	Ireland
	The Netherlands
	UK
Sojade	France
	Germany
	Ireland
	Italy
	Spain
	UK
Sojasun	France
The Coconut Collab	UK
Valio Oddly Good	Estonia
	Finland
	Latvia
	Poland
	Spain
	Sweden
Valsoia	Italy
Vive Soy	Spain
YoSoy	Spain

Supplementary Material. Table S3. Private (retail) brand labels of plant-based drinks and alternatives to yogurt across seven European countries included in our analysis

Country	Brand	No. of PBD samples	No. of PBAY samples	Total
**Belgium**	Boni	5	0	5
	Delhaize	2	2	4
	Everyday	1	0	1
	Belgium TOTAL	***8***	***2***	***10***
**France**	Carrefour	6	1	7
**Germany**	Edeka	11	2	13
	Kaufland K-TAKE IT VEGGIE	9	1	10
	Rewe - Bio Vegan	5	0	5
	Germany TOTAL	***25***	***3***	***28***
**Spain**	Carrefour	5	2	7
	Dia	1	0	1
	Lidl Vemondo	6	2	8
	Mercadona	3	0	3
	Spain TOTAL	***15***	***4***	***19***
**Sweden**	Coop	3	1	4
**The Netherlands**	Albert Heijn	5	3	8
**UK**	Asda	2	0	2
	Sainsbury's	6	0	6
	Tesco	6	0	6
	Waitrose	2	0	2
**UK Total**	***UK TOTAL***	***16***	***0***	***16***
**Grand Total**		**78**	**14**	**92**

PBD = plant-based drinks, **PBAY** = plant-based dairy alternatives
